# Acid-base status is an important factor for inflammation, but don’t forget CO_2_!

**DOI:** 10.1186/s13054-014-0664-0

**Published:** 2014-12-04

**Authors:** Didier Payen, Houda Haloui

**Affiliations:** Department of Anesthesiology & Critical Care & SAMU, Hôpital Lariboisière, AP-HParis, 2 Rue Ambriose Paré, 75010 Paris, France; Unité INSERM 1160, Université Paris 7 Denis Diderot, 1 rue Claude Vellefaux, 75010 Paris, France

## Abstract

Zampieri and colleagues used sophisticated statistical methods to create a picture of acid-base pattern and inflammation relationship in a clinical context. The observed independent relationship between acidosis and albumin concentration and inflammatory pattern opens up a new area for research. It has become clear that, in addition to the characterization of mediators, receptors, and cellular phenotypes, the inflammatory response has to be interpreted in light of acid-base status, albumin concentration, and probably also carbon dioxide level.

Until now, the interplay between acid-base status and inflammation has received little attention, especially in a clinical context. The article by Zampieri and colleagues [1] in a previous issue of *Critical Care* is a pioneering study analyzing the relationship between acidosis variables, inflammatory mediators, and end-organ failures (acute kidney injury and shock). Since the metabolic and inflammatory reactions are simultaneous, the demonstration of interplay that is more than a simultaneous modification remains a difficult challenge. Because of this, the authors used three different statistical methods to separate the confounding factors. First, they developed a generalized linear model using the measured mediator as a dependent variable and components of acid-base status as variables. Second, they performed a multivariate adaptive regression with splines in order to evaluate the association of selected cytokines and acid-base components. Third, they performed a principal component analysis using Simplified Acute Physiology Score 3 as a way of quantifying illness severity in order to assess the independent association of acid-base variables and cytokine levels. The authors found that, in 87 prospective unselected patients, the level of strong anion gap (SIG) was positively associated with TNFα and IL-6, IL-8, and IL-10. A negative association was found between albumin level and TNFα and IL-6, IL-7, IL-8, and IL-10 and IFNγ. The conclusion drawn from these results opens up a new route for research to understand the mechanisms that link acid-base variables, albumin level, and immunological activation.

Such a topic is important and clinically relevant since plasma and interstitial fluid constitute the microenvironment for immune and tissue cells. Acid-base and albumin characteristics may then interfere with the cell response to different signals such as endotoxin. In addition, both fluid resuscitation and capillary leak may largely influence the composition of the cell microenvironment, especially when a crystalloid such as saline or a balanced crystalloid such as Ringer’s lactate is used. The role of surrounding cell pH could be seen as a result of metabolic acidosis and carbon dioxide (CO_2_) level, an aspect that was not investigated in the study [2,3]. Given the picture presented in this article, some approaches might be tested to clarify the mechanisms involved in immune modifications induced by acid-base changes. First, immune cells should be drawn from septic patients that have been incubated in the septic plasma or drawn after replacement of septic plasma by healthy plasma; both acid-base conditions or albumin concentration can then be modified to test their impact on immune cells phenotype. This might help to clarify how the pH, the SIG, and albumin concentration change the immune cell phenotypes. Second, similar experiments with healthy cells incubated in plasma from acutely injured patients could be performed to demonstrate the role of physicochemical plasma patterns. Mediators and cell functions then could be evaluated in different acid-base conditions. Until now, few data on alkalosis have been reported in terms of immunity, and the essential information comes from acidosis situations. One author of the study was part of a group [4] that showed that metabolic acidosis induced by hydrochloric acid and lactic acidosis added to culture media of RAW 264.7 cells have opposite effects: hydrochloric acid at a pH of 7 seems essentially pro-inflammatory (nitric oxide level, IL-6/IL-10 ratio, NF-κB DNA binding), whereas lactic acidosis is essentially anti-inflammatory. A group with the same author, using a rat model, confirmed these results in terms of systemic cytokines [5]. In that study, the authors found a positive relationship between SIG and IL-6, IL-8, and IL-10 and TNFα, which was independent of illness severity. Even though albumin was not administered in the presented cohort, it can be discussed in light of immune effects. The authors observed a negative correlation between albumin level and IL-6, IL-8, IL-10, monocyte chemoattractant protein-1 and IFNγ. In addition to its complex effects, albumin was shown to be immunosuppressive for peripheral blood monocytes (IFNγ and TNFα) and also for T lymphocyte clone [6], which confirmed the presented results. Except for specific indications, albumin is not recommended for use in fluid resuscitation, especially after the recent negative results of a randomized clinical trial [7]. Third, the role of hypo- or hypercarbia has to be investigated since elevated CO_2_ was shown to modulate mammalian inflammatory and innate immune responses *in vitro* and *in vivo* [3], independently of extra- and intra-cellular pH. During a sterile insult of inflammation stimulation, hypercapnia may be of benefit but would be deleterious in the setting of infection due to host immunosuppression. The underlying mechanism implicates the NF-κB signaling pathway as an important hub of CO_2_ sensitivity [3,8]. This, in combination with the ability of elevated CO_2_ to enhance bacterial and fungal virulence and survival, suggests that hypercapnia may predispose humans to infections or worsen outcomes [3]. Understanding the involved molecular signaling pathways will be of great importance in the identification of new approaches to control infection and inflammation in the clinical setting.

## Abbreviations

CO_2_: Carbon dioxide
IL: Interleukin
IFNγ: Interferon-gamma
NF-κB: Nuclear factor-kappa-B
SIG: Strong anion gap
TNFα: Tumor necrosis factor-alpha
